# Pathological features, clinical presentations and prognostic factors of ovarian large cell neuroendocrine carcinoma: a case report and review of published literature

**DOI:** 10.1186/s13048-019-0543-z

**Published:** 2019-07-25

**Authors:** Xiaohang Yang, Junyu Chen, Ruiying Dong

**Affiliations:** 1 Department of Obstetrics and Gynecology, Qilu Hospital, Shandong University, Jinan, China; 2 Department of Obstetrics and Gynecology, Peking Union Medical College Hospital, Chinese Academy of Medical Sciences and Peking Union Medical College, Beijing, China

**Keywords:** Large cell neuroendocrine carcinoma, Ovarian tumor, Chromogranin A, Synaptophysin, Poor prognosis, Platinum-based chemotherapy

## Abstract

**Background:**

There is no consensus on the optimal chemotherapy regimen and the prognostic factors for ovarian large cell neuroendocrine carcinoma (LCNEC), a rare type of tumor. The objective of the present study is to present the case of a recent encounter of pure ovarian LCNEC and perform a brief review to summarize the clinicopathological features and prognostic factors of 57 cases of LCNEC patients that have been previously reported.

**Method: case presentation:**

Eligible studies were searched for online and 57 cases with clear follow-up data were found to have been reported. We present the 58th case, which is of a 70-year-old woman with stage IIIc primary pure LCNEC of the ovary. The initial symptom of this patient was abdominal distension (more than 2 months). A recent ultrasound test showed a solid-cystic mass occupying the pelvic and abdominal cavity. She received two courses of cisplatin-etoposide chemotherapy as an adjuvant therapy. No signs of nonclinical or radiological evidence of disease recurrence was found at follow-up examinations during the first 3 months after operation. A retrospective review of these 58 cases was conducted and survival curves were estimated. Using the Kaplan-Meier method.

**Conclusion:**

The patients included were aged between 18 and 80 years. A Kaplan-Meier survival curve revealed that the median overall survival was 10.000 months, while 26 (44.83%) patients died within 12 months. We compared the overall mean survival time of all patients with that of stage I patients (42.418 vs 42.047 months), which suggests that ovarian LCNEC has a very poor prognosis even at stage I. Mean survival was longer for patients who had undergone postoperative chemotherapy than for those without postoperative chemotherapy (48.082 vs 9.778 months). A small series, such as this, does not provide adequate data to establish a firm correlation between the postoperative chemotherapy and prognosis (*p* = 0.176). In our review of 58 cases with ovarian LCNEC, prognosis was unfavorable in most cases. Given the rarity of LCNEC, it is highly recommended that a global medical database of ovarian LCNEC and a standard system of diagnosis and treatment is established.

## Background

Large cell neuroendocrine carcinoma (LCNEC) of the ovary, a rare tumor that is often accompanied by other epithelial and germ cell tumors, is an extremely malignant tumor with an aggressive lethal outcome [1–3]. However, there are also some rare diseases entities related to the histology of pure large cell neuroendocrine carcinoma. According to the World Health Organization (WHO), primary ovarian LCENC is synonymous with undifferentiated type of non-small cell neuroendocrine carcinoma (NSNEC), possessing the characteristics of a large pleiomorphic nucleus with large round or oval nuclei and a tendency of neuroendocrine differentiation [3–6]. Additionally, assessment of neuroendocrine differentiation through immunohistochemical analysis, such as positive immunostaining for chromogranin A (CgA), synaptophysin (Syn) or Neural Cell Adhesion Molecule (NCAM, also the cluster of differentiation CD56), is required to confirm the diagnosis of LCNEC [5–8]. The initial symptoms presented by LCNEC of the ovary are identical to that of epithelial ovarian cancer (EOC), such as presence of an abdominal mass, pain or distention. Anderson Cancer Center reported on a total of 11 cases of NSCNEC from 1990 to 2005, with the most common symptoms being abdominal pain (6/11), ascites (2/11), pelvic mass (1/11), vaginal bleeding (1/11), and abdominal distension (1/11) [3].

The clinicopathological features of LCENC show that this type of tumor has an invasive clinical behavior and that the LCNEC components metastasize relatively early, affecting women of all ages (Table 1). To date, only 57 ovarian LCNEC cases with a definite follow-up period have been reported in the literature. Among a large number of Chinese and foreign literature, we found only 43 cases of ovarian tumor involving LCNEC together with surface epithelial stromal tumors and/or teratoma (Table 1). Additionally, only cases of 14 ovarian LCNEC patients without any associated components are detailed in the current range of cases (Table 1). Although progress has been made, despite extensive surgery and adjuvant chemotherapy, the biological aggressiveness and poor prognosis for this type of tumor common in the published literatures, even when diagnosis is made at an early stage [3, 6, 9]. Herein, we present a case of a 70 year old woman with stage IIIc primary pure LCNEC of the ovary and evaluate the clinicopathological features and prognostic factors of ovarian LCNEC using the data of these 58 cases.

**Table 1:** Clinicopathological features of 58 cases of original LCNEC

Ref	Age	Stage	Size (cm)/ Laterality	Associated component	Operation	Chemotherapy	Follow-up
1. [10]	27	Ia	11/left	None	LSO/ROVBx/OM/AP/PALA/peritoneal Bx	TC	NED10 m
2. [11]	64	Ia	14/right	None	TAH/BSO/OM	BEP	NED 9 m
3. [12]	73	IV	9/left	None	TAH/BSO/OM/nephrectomy/resection of brain metastasis	TC → γ -knife	NED 12 m
4. [6]	64	IV	11/right	None	BSO/TAH/OM/peritoneal Bx	TC (neo adjuvant)	NED 64 m
5. [13]	40	IIIc	15/left, 7/right	None	BSO/OM//PALA/bladder and sigmoid colon deposit excision	EP	NED 6 m
6. [14]	50	IV	25/left	None	BSO/TAH/partialOM/AP	TC	DOD 3 m
7. [15]	35	IIIc	6/left	None	TAH/BSO	No	AWD 3 m
8. [16]	44	IIIc	9/right	None	TAH/BSO/OM	Yes	DOD 22 m
9. [17]	76	IIb	35/eft	None	TAH/BSO/OM	No	DOD post op
10. [18]	77	IV	15/unclear	None	Palliative surgery	Etoposide&carboplatin	DOD 1.5 m
11. [18]	58	Ia	Unclear/left	None	TAH/BSO/LM/OM	TP → taxotere	DOD 17 m
12. [18]	67	III	13/left	None	TAH/BSO/LM/OM/PALA/peritoneal Bx	TC	NED 5 m
13. [19]	75	IV	13/right	None	TAH/BSO/LM/OM/PALA/peritoneal Bx	Etoposide&carboplatin →TC	NED 36 m
14. [20]	46	III	12/right	None	TAH/BSO/OM	No	DOD 4 m
15 present case	70	IIIc	20/right	None	TAH/BSO/OM	EP	NED 3 m
16. [21]	71	IIIb	6.5/right	Serous carcinoma	TAH/BSO	TC	NED 8 m
17. [22]	47	Ic	11/unclear	Serous adenocarcinoma & malignant brenner tumor	TAH/BSO/LM/OM/AP/PALA	TP	NED 24 m
18. [23]	68	IIIc	7/right, 5/left	Serous adenocarcinoma with focal mucin secretion	BSO/OM	TC → Doxil	DOD 7 m
19. [24]	44	Ia	25/left	Mucinous intraepithelial adenocarcinoma	TAH/BSO/OM	TC	DOD 4 m
20. [2]	45	Ib	18/right	Mucinous cystadenoma (BLM) & partial intraepithelial carcinoma	TAH/BSO/OM	Yes	DOD 36 m
21. [2]	58	IIIb	30/left	Mucinous cystadenoma (BLM) & partial intraepithelial carcinoma	TAH/BSO/OM/AP/LM/peritoneal Bx	Yes	DOD 8 m
22. [25]	34	Ic	16/left	Mucinous cystadenoma (BLM) & mucinous adenocarcinoma	TAH/BSO/OM	CP	DOD 8 m
23. [3]	22	I	21/right	Mucinous tumor (BLM) & mucinous adenocarcinoma	RSO/AP	CP	DOD 3 m
24. [4]	22	Ia	21/right	Mucinous cystadenoma (BLM) & mucinous adenocarcinoma	RSO/AP	CP	DOD 3 m
25. [3]	55	I	26/right	Mucinous tumor (BLM) & intraepithelial carcinoma	TAH/BSO	Pt-based chemotherapy	NED 68 m
26. [3]	55	III	13.5/right	Mucinous tumor (BLM)	TAH/BSO	Pt-based chemotherapy	DOD 2 m
27. [26]	18	IV	10/unclear	Mucinous tumor (BLM)	TAH/BSO/OM/LM/PALA /prior oophorocystectomy	TC	DOD7m
28. [3]	54	I	14/right	Mucinous & endometrioid carcinoma	TAH/BSO	Pt-based chemotherapy	NED 66 m
29. [9]	50	Ia	15/right	Mucinous adenoma	TAH/BSO/LM/OM	EP → TC	DOD 7 m
30. [27]	65	Ia	16.5/left	Mucinous adenoma	TAH/BSO/LM/OM/AP	No	DOD10m
31. [28]	35	Ic	Unclear	Mucinous adenoma	TAH/BSO/OM	Pt-based chemotherapy	NED 120m
32. [29]	68	IV	20/left	Adenocarcinoma	TAH/BSO/LM/OM	Etoposide&carboplatin, palliative radiation therapy	DOD 7 m
33. [3]	39	IV	5/right	Mucinous adenocarcinoma	TAH/BSO	Pt-based chemotherapy	AWD 8 m
34. [24]	73	IIIc	11/left	Mucinous micro invasive adenocarcinoma	BSO/OM/LM(Bx)/prior TAH	TP	DOD 8 m
35. [3]	59	I	14/ left	Adenocarcinoma, NOS	BSO	Pt-based chemotherapy	NED 28 m
36. [30]	35	III	15/right	Mucinous cystadenoma (BLM) & mucinous adenocarcinoma	TAH/BSO/OM/AP	Yes	DOD 4 m
37. [31]	40	Ia	30/left	Mucinous adenoma, cystadenoma (BLM) & mucinous adenocarcinoma	TAH/BSO/LM/OM/AP/PALA	Yes	NED 8 m
38. [32]	21	Ia	5/right	Mucinous tumor (BLM)	RSO	BEP → TC	DOD 5 m
39. [33]	70	Ia	16/right, unclear/left	Mucinous tumor (BLM)	TAH/BSO/LM/OM/AP	No	NED 6 m
40. [34]	48	I	15/right	Mucinous cystadenoma (BLM) & mucinous adenoma	TAH/BSO/LM/OM	No	NED 3 m
41. [35]	36	IIIc	26/right	Mucinous endocervical tumor (BLM)& intraepithelial carcinoma	TAH/BSO/LM/OM	Yes	NED 6 m
42. [6]	65	Ic	15/left	Endometrioid adenocarcinoma & squamous differentiation mucinous adenocarcinoma	TAH/BSO/OM	TC	DOD 2 m
43. [6]	80	IIc	7/left	Endometrioid adenocarcinoma	TAH/BSO/PLA/OM/AP	TC	NED 40 m
44. [6]	42	IIIb	13/left, unclear/right	Endometrioid adenocarcinoma	TAH/BSO/PLA/OM/PALA	TC	NED 32 m
45. [2]	77	Ia	15/unclear	Endometrioid adenocarcinoma	TAH/BSO/LM/peritoneal Bx	No	DOD 19 m
46. [36]	33	Ic	11/left	Endometrioid adenocarcinoma	LSO/ROVBx/partial OM	Camptosar	DOD 6 m
47. [3]	53	III	14.5/left	Endometrioid adenocarcinoma	TAH/BSO	Pt-based chemotherapy	NED 37 m
48. [3]	63	IV	14/left	Endometrioid adenocarcinoma	TAH/RSO	Pt-based chemotherapy	DOD 9 m
49. [37]	49	III	4.6/left	Endometrioid adenocarcinoma	TAH/BSO/LM/OM/peritoneal Bx	Yes	NED 4 m
50. [38]	53	I	21/left	Mucinous tumor (BLM), invasive mucinous adenocarcinoma & teratoma	TAH/BSO/LM/OM	EP	DOD 7 m
51. [38]	53	IV	20/left	Mucinous tumor (BLM), invasive mucinous adenocarcinoma & teratoma	TAH/BSO/LM/OM/peritoneal Bx	TC	DOD 5 m
52. [3]	47	III	14/right	Adenocarcinoma, teratoma & NOS	TAH/BSO	Pt-based chemotherapy	NED 11 m
53. [28]	56	IIc	18/right	Mucinous carcinoma & teratoma	TAH/BSO/peritoneal Bx/PLA	No	DOD 10 m
54. [16]	56	Ic	18/right	Mucinous adenocarcinoma & teratoma	TAH/BSO/LM	No	DOD 10 m
55. [3]	25	IV	5/right	Teratoma	BSO/OM/AP	Pt-based chemotherapy	DOD 36 m
56. [39]	69	IV	15/left	Teratoma	LSO/thoracic Bx	TC	DOD 6 m
57. [39].	54	III	Unclear	Teratoma	TAH/BSO/ sigmoid resection	Yes	NED 12 m
58. [3]	42	IV	Unclear	Benign cyst & teratoma (contralateral ovary)	TAH/BSO	Pt-based chemotherapy	DOD 20 m

## Case presentation

We report the case of a 70 year old woman with no clear trigger, who presented herself with abdominal distension (more than 2 months). A recent ultrasound test revealed an 18 cm solid cystic mass occupying the pelvic and abdominal cavity with rich intralesional vascularization. Her cancer antigen 125 (CA125) level was relatively high at 367.90 U/ml, neuron-specific enolase (NSE) and fragment of human cytokeratin 21–1 (CYFRA21-1) levels were elevated to 24.83 and 3.85 ng/ml, respectively.

A laparotomy was carried out and 0.5 l of hemorrhagic ascitic fluid was drained. During the procedure, we found a 20 cm diametric cystic and solid right ovarian mass, which had burrowed into the uterus, intestinal tube and parietal pelvic wall. Metastatic lesions had spread diffusely throughout the peritoneum and the surface of the uterus and intestinal tube. There were no obvious abnormal changes in the right ovary and oviduct, pelvic lymph node and para-aortic lymph node. A right salpingo-oophorectomy was performed, and intraoperative frozen section consultation showed a poorly differentiated carcinoma, and therefore a total abdominal hysterectomy with left salpingo-oophorectomy, omentectomy, along with removal of pelvic metastases was conducted. General observation of the samples displayed a right ovarian tumor measuring 33 × 23 × 5 cm, whose lesions were very fragile with a nodularity like rotten flesh surface, and its cut section showed a gray white focus and partial hemorrhage and a necrosis area. The most conspicuous pelvic metastases mass was 12 × 10 × 3.5 cm with an irregular and dusty pink external surface and the section cut showed cystic and hemorrhagic areas.

**Fig. 1:**
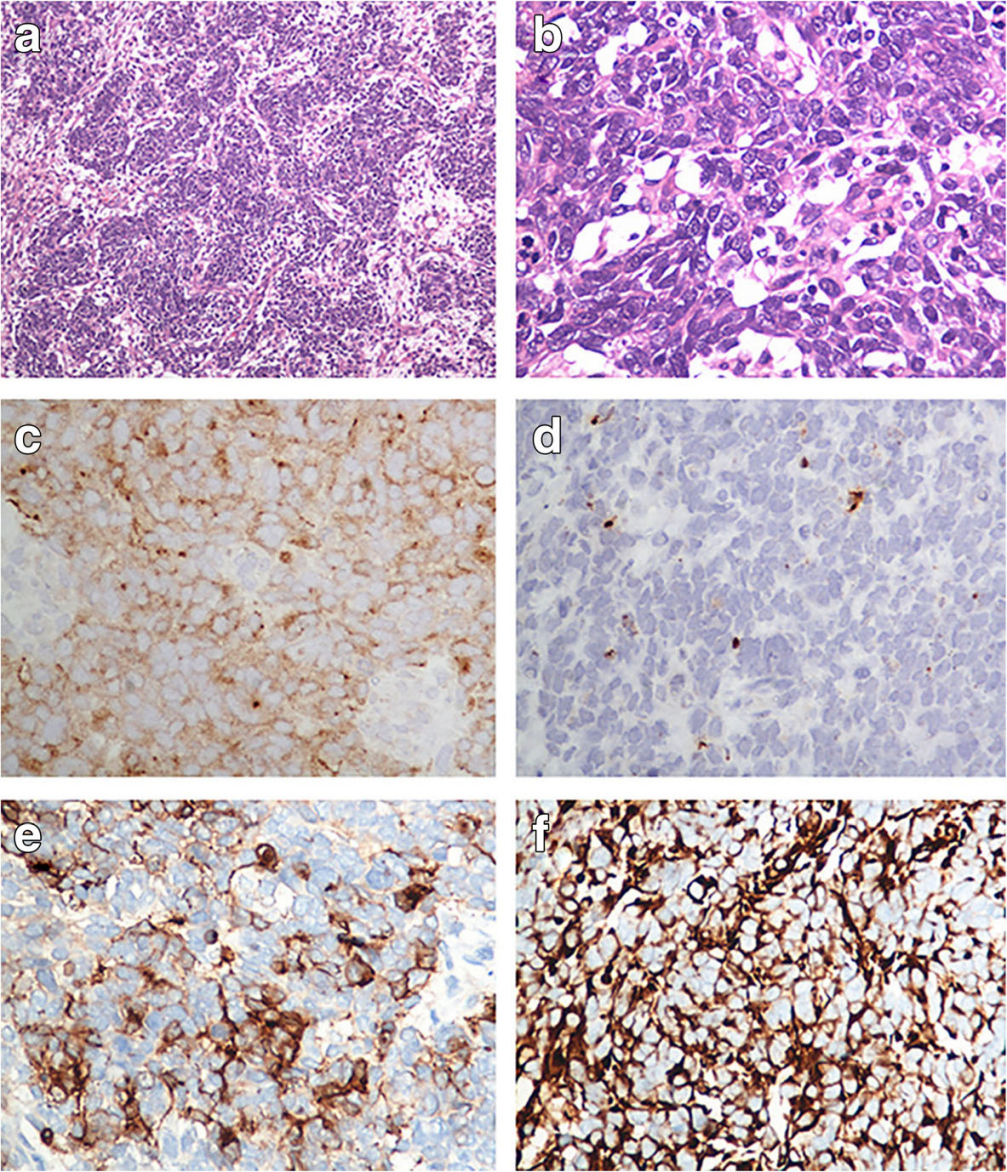
Neoplastic cells were compactly arranged in rosette-like and trabecular patterns (**a**), tumor cells with obvious nuclei, granular chromatin and numerous mitotic activities (**b**), neuroendocrine carcinoma focally positive for Syn (**c**), CgA (**d**), CD56 (**e**) and Vimentin (**f**)

The pathology of this original surgery was interpreted as poorly differentiated large cell neuroendocrine carcinoma of the right ovary with the involvement of metastasis lesions on the surface of the oviduct, partial perimetrium and pelvic area. When the H&E stained slides were observed under the microscope, the predominant pattern of lesions comprised mostly of trabecular and rosette-like formations, surrounded by connective tissues at the periphery. Pleomorphic hyper-chromatic tumor cells were arranged in rosette-like patterns, and frequently showed a high mitotic rate (Fig. 1a). The tumor cells had large, moderate amounts of cytoplasm and round to oval nuclei, occasionally with conspicuous nucleoli, granular or coarse chromatin (Fig. 1b). Immunohistochemistry (IHC) was performed in order to confirm the ultimate histological diagnosis. In the areas of neuroendocrine components, the tumor cells were positive for Syn, cytokeratin (CK), Wilms’ tumor suppressor gene (WT-1) and Vimentin. The tumor cells were also diffusely and intensely positive for CgA and PAX-8, with focal and intense staining for CD56 and EMA. While immunostaining for ER was negative (Fig. 1c, d, e and f). A definite diagnosis of ovarian LCNEC (International Federation of Gynecology and Obstetrics stage IIIc; American Joint Committee on Cancer staging T3cN0M0) was made based on the clinical presentation, histopathological features, and IHC profiles.

The patient received 3 cycles of postoperative adjuvant chemotherapy consisting of 120 mg/M2 Etoposide from day 1 to day 5 and 100 mg/M2 Cisplatin on day 1. After 3 months of follow-up, she was alive with no clinical or ultrasonographic evidence of disease recurrence.

## Methods

We screened for potentially eligible titles, using combinations of the following keywords: (“large cell neuroendocrine carcinoma” or “non-small cell neuroendocrine carcinoma” or “LCNEC” or “NSCNEC” or “NSNEC”) and (“tumor” or “cancer” or “carcinoma” or “neoplasm” or “malignancy”) and (“ovarian” or “ovary”) and (“survival” or “outcome” “prognosis” or “prognostic” or “mortality”) between Jan 1, 1990 and Aug 29, 2018 in the PubMed database, ClinicalTrials.gov, China National Knowledge Infrastructure (CNKI) database and Wanfang Med Online. We identified 58 cases with explicit follow-up periods, the data for which is summarized in Table 1 (including our present case). A limited number of available cases were reviewed to provide the characteristics of LCNEC and identify prognostic factors. Statistical analysis was performed using the R Programming Language. Survival curves were compared using Kaplan-Meier method. For all statistical tests, the differences were considered as statistically significant when the *p* value was < 0.05.

## Discussion

### Origins

The histogenesis of neuroendocrine tumors is currently unclear. The following hypotheses have been proposed regarding the origins of LCNEC:

1. **Derived from neuroendocrine cells:** Neuroendocrine cells are distributed in the normal epithelium of benign, borderline, and malignant tumors of the female genital tract. These mature neuroendocrine cell components serve as an origin of neuroendocrine tumors of the ovary through neoplastic transformation [2, 25, 35].
2. **Derived from non-neuroendocrine cells:** Ovarian neuroendocrine tumors may be transformed from non-neuroendocrine cells through neoplastic neuroendocrine transformation that occurs along with the activation of gene sequences, similar to that of neuroendocrine cells [21, 25, 35, 38, 40, 41].
  The first two hypotheses may better explain the fact that most ovarian LCNECs are regularly associated with other surface epithelial tumors.
3. **Derived from teratomatous cells:** There is another hypothesis as that LCNEC originates from teratomatous cells. This hypothesis is based on the fact that ovarian carcinoids are frequently accompanied by teratomas as well but are rarely associated with surface epithelial tumors.
  However, the association between a LCNEC and a pure teratoma is uncommon and only three cases have been reported in the literature, therefore this is unsubstantiated hypothesis. There are 4 cases of ovarian LCNEC with the combination of teratoma and other epithelial tumors in the published literature. For teratoma, the tip of the occurrence of a mucinous tumor is followed by focal dedifferentiation into a LCNCE, which may explain this peculiar combination [38].
4. **Derived from primitive cells:** Primitive endocrine cells or common stem cells capable of multidirectional differentiation can differentiate into both endocrine and other cell types [25, 35, 38]. The last hypothesis is in favor of the existence of pure ovarian LCNEC, which is a direct result of normal ovarian tissue and is further supported by the fact that isolated neuroendocrine cells have been identified in normal ovaries [42, 43].

### Differential diagnosis

From a practical standpoint, LCNEC does not have specific radiological features. Therefore, it is far from adequate for diagnosis to be made merely through imaging findings alone [10, 21]. Additionally, differentiation between benign and malignant processes through radiological inspection is unreliable. Computed Tomography Scan (CT) has advantages over ultrasound imaging for the exploratory inspection of mesenteric or peritoneal thickening. Emission Computed Tomography (ECT) can distinctly show multiple small nodules that distribute peritoneum, mesentery and omentum. Ultrasonography can indicate a mass of abdominal effusion. Laparoscopic surgery will allow for an assurance of the definitive diagnosis, due to lower invasiveness.

Other primary or secondary neuroendocrine tumors of the ovary, such as primary or metastatic carcinoid tumor, small cell carcinoma (SCC) of the pulmonary or hypercalcemic type, metastatic neuroendocrine carcinoma, are included in the distinguished diagnosis [14, 15, 17, 18, 23, 30]. Given that some non-neuroendocrine tumors may show neuroendocrine differentiation, teratoma, sex-cord stromal tumor and Sertoli-Leydig cell tumor, should also be included in the differential diagnosis. The main differential diagnoses are summarized in Table 2.

**Table 2:** Differential diagnosis and pathological features of LCNEC

Differential diagnosis	Pathological features
LCNEC	Solid nests or trabecular patterns, large tumor cell with high mitotic rate [3, 6, 20]. Positive reactivity for CgA, Syn, CK and CD56 [5, 6, 8, 11, 40, 44].
Primary or metastatic carcinoid tumor	Uniform cell with rare mitotic figure and inexistent necrosis [17].
SCC pulmonary type	Smaller cell with obvious necrosis and less-intense immunohistochemical reactivity for CK and CgA^.^ [30].
SCC hypercalcemic type	Follicle-like spaces,large cells with pale intracytoplasmic hyaline globules [10, 45, 46] and clinical manifestations of hypercalcemia [30].
Metastatic neuroendocrine carcinoma	Bilateral ovarian involvement, vascular invasion and inmiscibility with epithelial layer of the ovary [23, 30].
Non-neuroendocrine tumors with neuroendocrine differentiation	Identifying non-neuroendocrine components of teratoma, sex-cord stromal tumor and Sertoli-Leydig cell tumor^.^[3, 10, 15, 17, 25].

### Clinical feature summary and prognostic factor analysis

Based on previously published reports, only 58 original ovarian LCNEC cases, including our current case, have been described in detail, and a summary of the clinicopathological features of these ovarian LCNEC cases are presented in Table 1.

These 58 patients were aged from 18 to 80 years and a total of 25 (43.10%) patients were of a childbearing age. About half of the patients in our study presented with an advanced stage disease (31 were FIGO stages III or IV; 24 cases of stage I; three cases of stage II). Of the 58 cases, only 15 cases were of pure neuroendocrine carcinoma, while the epithelial or germ cell components in 43 cases of ovarian LCNEC included mucinous tumors (benign, borderline malignant and malignant), endometrioid adenocarcinomas, mature cystic teratomas, adenocarcinomas, serous adenocarcinomas and benign ovarian cysts.

**Fig. 2:**
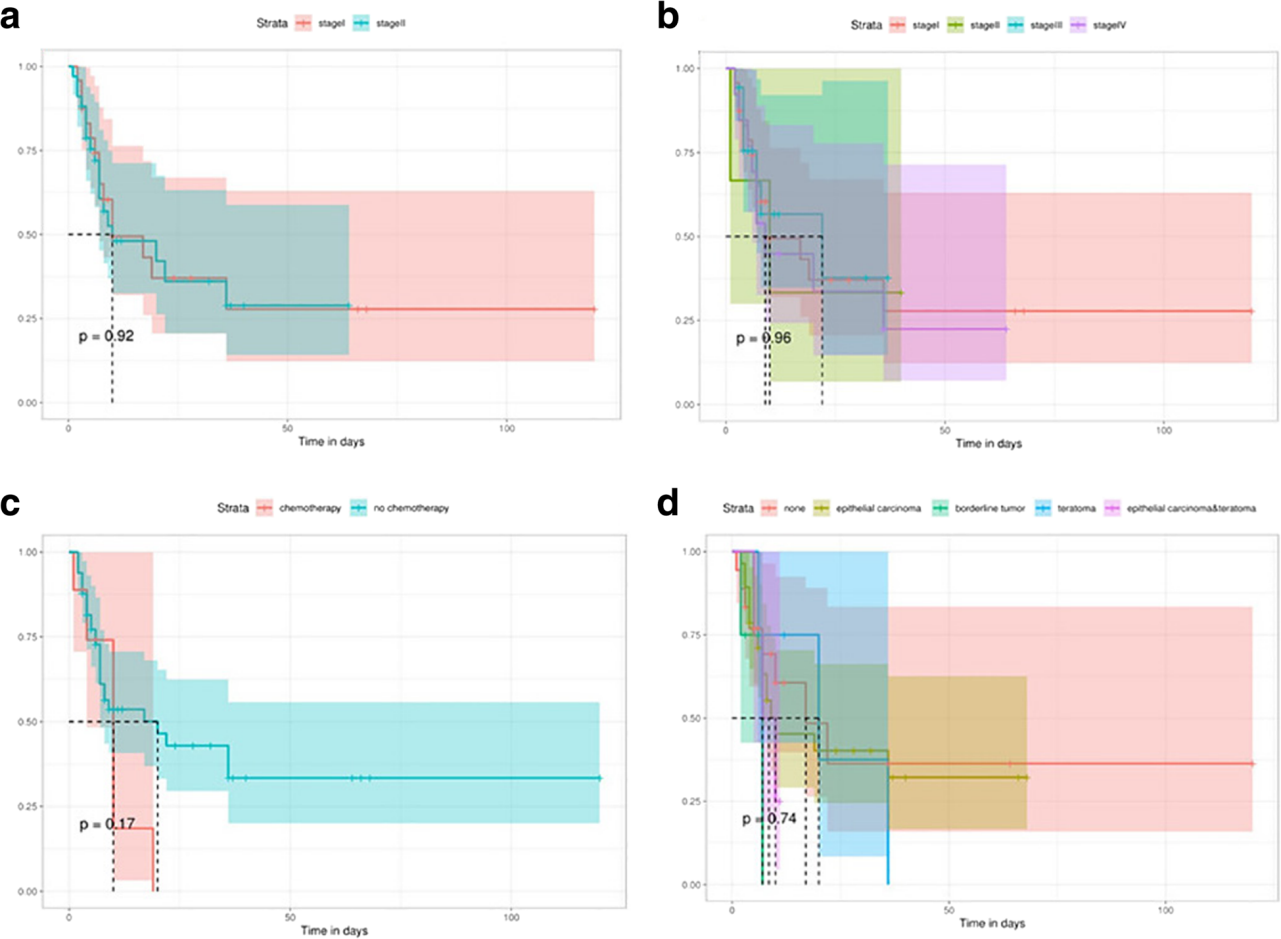
Overall survival Kaplan-Meier curves were compared between patients in stage I with those in stage II,III,IV (**a**), overall survival Kaplan-Meier curves were compared for each stage (**b**), overall survival Kaplan-Meier curves were compared between patients who underwent postoperative chemotherapy with those who did not (**c**), overall survival Kaplan-Meier curves were compared for various pathological types (**d**)

In the 49 cases described, for which the patients had undergone adjuvant chemotherapy treatment; platinum-based chemotherapy accounted for a majority of the cases. However, the prognosis of ovarian LCNEC is recognized to be extremely poor despite extensive surgery and adjuvant chemotherapy. A Kaplan-Meier survival curve of the 58 cases, based on data in the published literature is shown in Fig. 2. It reveals that median overall survival is 10.000 months, while 26 (44.83%) patients died within 12 months. In particular, a patient with a combination of ovarian LCNEC and a mucinous adenoma endured nearly 120 months of survival under the use of platinum-based chemotherapy. It appears that this is an isolated case, but it is useful to remember that the long-time disease-free survival of some of the patients may be related to the use of platinum-based chemotherapy. The average overall survival time was 42.418 months for all stages, and only 42.047 months for stage I cases, which suggests that LCNEC of the ovary has a very poor prognosis even at stage I (Fig. 2a). Patients who were of a higher FIGO stage didn’t have a better prognosis, compared with that of the others. The survival curves for each stage had route near-overlap, which suggests that postoperative pathological staging is hardly correlated with prognosis (Fig. 2b).

As indicated by Veras et al., certain patients may have a more favorable prognosis, particularly those at stage I and/or those who have received platinum-based therapy [3]. Therefore, we compared the mean survival time of ovarian LCNEC with postoperative chemotherapy (48.082 months) with ovarian LCNEC without chemotherapy (9.778 months) using a Kaplan–Meier curve that was based on the results of the published studies. Unfortunately, such a small series like this does not afford for a firm correlation between chemotherapy and prognosis (*p* = 0.176), as shown in Fig. 2c. Although the data suggests that that platinum-based chemotherapy may be the optimal chemotherapy regimen for LCNEC, no consensus exists. Moreover, we tried to figure out the correlation between pathological classification and prognostic evaluation in these 58 cases and define it in a clearer manner, but we didn’t have much success. We were unable to determine the influence of tumor pathological type on prognosis based on the very small number of cases (Fig. 2d).

Furthermore, previous studies have found that the overexpression of synaptophysin is an independent contributory factor for undesirable prognosis, through a multivariate analysis (HR = 10.82, 95% confidence interval 3.10–37.71, *p* < 0.0001), and that it is not related with age, FIGO stage or residual tumors after surgery [47]. That is, a high proportion of neuroendocrine components might result in dismal prognosis of ovarian high-grade serous carcinomas. Therefore, explicitly confirming the proportion of neuroendocrine components can be used for the pathological diagnosis of ovarian LCNEC. If there are a high proportion of epithelial elements in mixed epithelial and large cell neuroendocrine ovarian tumors, the optimal treatment should be used against the epithelial component, such as first-line chemotherapeutic regimens of paclitaxel plus carboplatin. In the case of pure LCNEC, direct consideration should be given to a platinum-etoposide chemotherapy regiment, aimed at neuroendocrine components [41]. Additionally, various combination chemotherapy regimens, such as platinum, paclitaxel, etoposide and bleomycin have been used in previous studies, while only one case used radiation therapy (Table 1).

A patient with LCNEC of the ovary associated with deleterious Breast Cancer Susceptibility Gene 2 (BRCA2) germline mutation was presented by Herold et al. BRCA1/2 mutation testing is a reasonable step for LCNEC patients to take, since they may benefit from targeted therapy with poly (ADP-ribose) polymerase inhibitors [19].

A total of 7 LCNEC patients were younger than 40 years of age and presented with stage I of the disease, with 5/7 patients undergoing a preservative operation of fertility and postoperative chemotherapy. Recently, a raft of studies have suggested that stage Ic and grade 3 are two independent predictors of survival and that it is associated with a significantly higher rate of distant recurrence, and that the type of surgical approach did not affect survival in EOC [48–51]. For some young women with early stage ovarian cancer, fertility-sparing surgery can be safely proposed.

However, no consensus has yet been reached in the available guides. Based on the most recent European Society for Medical Oncology (ESMO) guidelines, conservative surgery can be applied to those with grade 1/2 Ia and Ic epithelial ovarian cancer with unilateral involvement and favorable histology (mucinous, serous, endometrioid, or mixed histology). For these types of patients with stage Ia of the disease, grade 1 and nonclear cell histology, surgery alone is adequate [52, 53]. Given the high risk of local and distant relapses, for women with stage Ic and grade 2/3, adjuvant chemotherapy should be considered after adequate surgery and staging [52]. According to the 2017 guidelines of the National Comprehensive Cancer Network (NCCN), unilateral salpingo-oophorectomy along with comprehensive staging can be considered for all grades Ia or Ic for EOC in the patients who desires to preserve fertility [53]. Fertility-sparing surgery is not recommended for women with stages II–III ovarian cancer, given the high recurrence and mortality rates for these women. Therefore, for these women, radical surgical treatments should be the standard [54].

The damage to the ovary relates to surgical, chemotherapy and radiation treatments for women with gynecological cancer, especially chemoradiotherapy. The extent of the damage to the ovary depends on many factors, the most important of which is chemotherapy type and dose [55], the ovarian reserve before treatment [56], as well as the dose, fractionation scheme and irradiation field of radiotherapy [55, 57]. Most ovarian cancer patients receive adjuvant chemotherapy after surgery. Anti-Mullerian Hormone (AMH) has emerged as a sensitive predictor of ovarian function [58], with a substantial reduction of AMH concentrations in peripheral blood being detected after months of chemotherapy, while circulating AMH concentrations may indicate the amount of ovarian damage [59, 60]. In order to preserve the fertility of patients undergoing postoperative adjuvant chemoradiotherapy, the general method in routine reproductive clinical practice is oocyte and embryo cryopreservation and ovarian transposition (oophoropexy), which at present can be offered to women undergoing pelvic irradiation [61].

Therefore, the studies of fertility preservation for EOC will be helpful in offering a similar method for LCNEC. However, we need to collect and analyze clinicopathological data, in order to formulate a standard for the management of fertility preservation surgery in reproductive-aged patients with stage I ovarian LCNEC. Based on recent research, cancer is known to influence survivors’ sexual function, motivation for childbearing and partner-related fears [62, 63]. A multidisciplinary team including oncology and reproductive endocrinology providers, as well as good communication between the team and psychosocial supporters about fertility preservation, is critical for women who undergo gonadotoxic treatments [56, 64].

A total of 12 LCNEC patients were older than 60 years of age and presented with stage III/IV of the disease, with 1/12 patients undergoing neoadjuvant chemotherapy. One of the oldest stage IV patients underwent palliative surgery and postoperative chemotherapy. Unfortunately, the patient died of the disease 1.5 months after surgery. Some elderly women with advanced ovarian LCNEC, are very fragile and with a lower life expectancy. Further trials are still required to determine whether standard treatment is of clinical benefit. This review of studies indicates that, in spite of a higher incidence of side effects among those who have received standardized treatment, elderly patients have benefited by being able to manage their gynecological cancers [65, 66].

Robotic-assisted surgical staging seems to be successful in patients with presumed early stage ovarian cancer that is associated with a minimal complication rate [67]. However, the use of robotic surgery for advanced ovarian cancer is limited and needs to be prospectively validated [68]. The data that we have collected, indicates that robotic-assisted surgery has not yet been used in treatment of ovarian LCNEC, but using robotic surgery in patients with early-stage LCNEC of the ovary may be the way forward. Enhanced Recovery After Surgery (ERAS) has been widely used in gynecological oncology treatment, and has given rise to a large number of benefits, such less complications and reduction in the length of hospital stay [69]. However, since implementation of ERAS requires cooperation among multiple fields, but it has seldom been reported in the therapy of ovarian LCNEC.

## Conclusion

In conclusion, ovarian LCNEC is uncommon and is defined as an extremely malignant type of tumor. Prognosis is poor even if early diagnosis is made. Therefore, it is highly recommended that LCNEC is differentiated from other ovarian tumors using histological features and immunohistochemical specificity. Multiple literature indicates that most patients undergo platinum-based postoperative chemotherapy, while there is still no convictive peroration on the prognosis as a result of the use of platinum-based chemotherapy. Due to the rarity of ovarian LCNEC and undisciplined follow-up, the effect of chemotherapy on long term survival has not been reported.

It is highly recommended that a global medical database of ovarian LCNEC be established, in order to collect and analyze inter-institutional clinicopathological data, in order that data on these types of rare tumors are discussed and shared at oncology conferences. We should proceed to carry-out a retrospective survey to elucidate the prognostic factors and identify prospective clinical studies that can be done to obtain further knowledge on the clinical characteristics and the biological behavior of ovarian LCNEC, using animal models to establish optimal therapeutic guidelines for these tumors.

## Data Availability

The datasets used during the current study are available from the corresponding author on reasonable request.

## Abbreviations

AMH: Anti-Mullerian Hormone
AP: Appendectomy
AWD: Alive with disease
BEP: BLM (bleomycin) / CHOEP (etoposide) /PDD (cisplatin)
BLM: Borderline malignancy
BRCA2: Breast Cancer Susceptibility Gene 2
BSO, RSO and LSO: Bilateral, right and left salpingo-oophorectomy
Bx: Biopsy
CA125: Cancer antigen 125
CgA: Chromogranin A
CK: Cytokeratin
CNKI: China National Knowledge Infrastructure
CP: CYC (cyclophosphamide) /PDD (cisplatin)
CT: Contrast-enhanced computed tomography
CYFRA21-1: A fragment of human cytokeratin 21–1
DOD: Died of recurrent disease
ECT: Emission Computed Tomography
EOC: Epithelial ovarian cancer
EP: CHOEP (etoposide) /PDD (cisplatin)
ERAS: Enhanced Recovery After Surgery
ESMO: European Society for Medical Oncology
IHC: Immunohistochemistry
LCNEC: Large cell neuroendocrine carcinoma
LM: Lymphadenectomy
NCAM: Neural Cell Adhesion Molecule (also the cluster of differentiation CD56)
NCCN: National Comprehensive Cancer Network
NED: No evidence of disease
NOS: Not otherwise specified
NSE: Neuron-specific enolase
NSNEC/NSCNEC: Non-small cell neuroendocrine carcinoma
OM: Omentectomy
PALA: Para-aortic lymphadenectomy
PLA: Pelvic lymphadenectomy
Pt-based CT: Platinum-based chemotherapy
SCC: Small cell carcinoma
Syn: Synaptophysin
TAH: Total abdominal hysterectomy
TC: PTX (paclitaxel) /CBP (carboplatin)
TP: PTX (paclitaxel) /PDD (cisplatin)
WT-1: Wilms’ tumor suppressor gene
